# ChatGPT, GPT-4, and Other Large Language Models: The Next Revolution for Clinical Microbiology?

**DOI:** 10.1093/cid/ciad407

**Published:** 2023-07-03

**Authors:** Adrian Egli

**Affiliations:** Institute of Medical Microbiology, University of Zurich, Zurich, Switzerland

**Keywords:** chatGPT, large language model, artificial intelligence, chatbot, digitalization

## Abstract

ChatGPT, GPT-4, and Bard are highly advanced natural language process–based computer programs (chatbots) that simulate and process human conversation in written or spoken form. Recently released by the company OpenAI, ChatGPT was trained on billions of unknown text elements (tokens) and rapidly gained wide attention for its ability to respond to questions in an articulate manner across a wide range of knowledge domains. These potentially disruptive large language model (LLM) technologies have a broad range of conceivable applications in medicine and medical microbiology. In this opinion article, I describe how chatbot technologies work and discuss the strengths and weaknesses of ChatGPT, GPT-4, and other LLMs for applications in the routine diagnostic laboratory, focusing on various use cases for the pre- to post-analytical process.

## Teaser

Natural language processing–based computer software simulates and processes human conversation. These potentially disruptive large language model technologies have a broad range of conceivable applications in medical microbiology. Strengths and weaknesses for applications in routine diagnostics are discussed.

In our information-centered age, large amounts of healthcare data are produced and interpreted by human experts. However, a lot of the data collected routinely in laboratories are not accessible or are underused, even though they may contain information of value to clinicians for patient management. Rather than wasting this information, how can we best use routine data to improve diagnostics and the advice provided by physicians? Improving the efficiency of data management and developing artificial intelligence (AI) tools capable of extracting maximum value from the extensive datasets now available will be vital for us to move into a knowledge-centered world [1].

OpenAI was founded with the goal of developing general AI to benefit society and be an “extension of human wills and, in the spirit of liberty, as broadly and evenly distributed as possible…”. OpenAI recently released a series of groundbreaking tools. Headlines were generated in mid-2022 with DALL•E 2, a revolutionary image generator, but without any obvious scientific application. Since then, even more powerful image generators with improved image quality have been released by smaller companies such as Midjourney and Stable Diffusion. Eventually, such technology could be used to generate images for medical training. However, at this stage, most science-related content is low quality. More recently, OpenAI launched ChatGPT (GPT-3.5) [2] and the newer GPT-4 [3], both “large language models” (LLMs) that show far greater potential for healthcare and laboratory applications (see Supplementary Table 1 for a list of recently launched LLM tools).

ChatGPT, GPT-4, Bard, and Claude are advanced chatbots that allow users to ask questions on an interactive level. Since launching, ChatGPT has garnered intense media coverage and amassed millions of subscribers. ChatGPT's language models were trained on billions of diverse Internet text files, including articles, forums, and websites, and fine-tuned on other datasets, including books and articles. This enabled ChatGPT to score 60.2% on a set of multiple-choice questions based on the US Medical License Exams, while Google's Med-PaLM 2 scored up to 86.5% [4]. Conceivably, in a few years, the introduction of ChatGPT may be regarded as the dawn of a new technological age for knowledge sharing [5], potentially as revolutionary as Gutenberg's printing press. It should also stimulate debate on the technology's profound impact on society, its potential to cause harm, the effects on job security and social inequity, and the problem of fake content generation in science and healthcare [3].

Technological revolutions often encounter resistance in their infancy, even among the scientifically literate. But what are the opportunities and threats from these technologies for clinical microbiologists? How will they affect routine practice in diagnostics? In this Opinion article, I explore the functionalities of chatbots, address technical requirements, provide use cases, and discuss applications in the pre- to post-analytical workflow.

## HOW DO CHATBOTS WORK?

Chatbots are computer programs designed to simulate human conversation to answer questions, provide customer service, and complete tasks [6, 7]. They are often integrated into messaging services, smartphone apps, and websites. There are several different approaches to building a chatbot, including rule-based systems, decision tree models, and machine learning [8, 9]. Rule-based systems rely on a predetermined set of rules to respond to user input [10, 11]. Decision tree models use a tree-like structure (eg, random forest) to determine the appropriate response based on user input [12]. Machine learning–based models use algorithms to learn from data and make decisions based on that learning experience [9]. Natural language processing (NLP) techniques are common for building machine learning–based chatbots [13, 14]. NLP models can analyze and understand human language to generate human-like responses [15, 16]. Supplementary Table 2 and Supplementary Text 1 explain how NLPs and LLMs work. NLP algorithms can be combined with reinforcement learning [17] to train them to take actions to maximize a reward [18]. As seen with ChatGPT and GPT-4, in some instances, an NLP model is trained to generate responses to user inputs, and a reinforcement learning algorithm is used to adjust the model to maximize the likelihood of generating appropriate and coherent responses. NLP-based chatbots can adjust their responses in real time based on the user's feedback and interactions.

Chatbots have the potential to interact with and assist clinical microbiologists, infectious diseases experts, and nurses in decisions regarding diagnostic tests and improving user interactions with medical microbiology laboratories. Autonomous AI agents such as AutoGPT and Baby-AGI can already interact with each other for problem-solving and could potentially be trained to independently access the literature to understand medical problems. Graph AI techniques that integrate diverse modalities and exploit cross-modal dependencies through geometric relationships are used to navigate multimodal, complex healthcare data, creating a “map” of interconnected data points that reveal how different types of data relate to and influence each other. This holistic view of a dataset allows more robust predictions and decision-making [19]. In medical microbiology, graph AI techniques could be leveraged to integrate patient data, including medical histories, current symptomatology, laboratory test results, and medical imaging data. By creating a network of heterogeneous data points, graph AI can discern patterns and correlations between seemingly disparate pieces of information, potentially leading to more accurate diagnoses and targeted treatment plans. This approach has already been applied to coronavirus disease 2019 (COVID-19) [20], tuberculosis, and antimicrobial resistance [21]. In a graph AI environment, LLMs provide the necessary help to access and interact with graph AI.

## WHAT MEDICAL CHATBOTS ARE AVAILABLE?

Where applied in medicine, chatbots provide healthcare-related information and support to users on topics such as COVID-19, weight loss, smoking cessation, and mental health (see Supplementary Table 3).

### Performance

While there is a range of applications in healthcare, they generally lack independent validation, and few randomized controlled trials (RCTs) using medical chatbots have been performed. Of the RCTs that have been conducted, most tested chatbot capacity to diagnose or treat mental health disorders. For example, patients using Woebot, a US Food and Drug Administration (FDA)–approved class II medical device, exhibited significantly reduced symptoms of depression over the study period compared with a control group [22].

Symptom checkers use machine learning algorithms to provide personalized health recommendations based on symptoms and other user information provided [23]. For infectious diseases, studies that use symptom checkers have focused on triaging COVID-19 [14, 24]. Ada Health is a symptom checker with class II certification under the EU medical device regulation. Ada's diagnostic accuracy for inflammatory rheumatic diseases and abdominal pain was significantly higher than physicians' (70% vs 54%) and comparable to the gold standard discharge rheumatologists’ diagnosis [25, 26]. In another study, patients self-assessed abdominal pain symptoms using Ada and were subsequently assessed by physicians. Ada suggested the final discharge diagnosis in 52% of cases, which was significantly lower than the diagnosis based on doctor–patient interaction (81%). However, when used together, Ada significantly increased the accuracy rate (87%) compared with the physician alone [26]. Despite their promise, only 2 studies have compared the performance of symptom trackers against each other, with both studies finding that the superior app was from the company that funded the study [24, 27].

Recently, ChatGPT has been assessed for its ability to provide quality and empathetic responses to patient questions. Chatbot responses were not only significantly longer than physician responses (211 words [interquartile range, 168–245] vs 52 words [interquartile range, 17–62]) but were also rated as higher quality and more empathetic [28].

### Regulations

The FDA and the European Union have complex regulatory frameworks for digital health technologies, including chatbots and symptom checkers [29, 30]. Supplementary Text 2 provides an overview of current regulatory elements that use this technology in medical applications (Supplementary Text 2). There are limited guidelines on how to evaluate, benchmark, and quality control the use of chatbots for medical purposes [31]. Most chatbot and symptom checkers remain a black box, with limited or no technical details available on which machine learning models are used and on how they were trained and validated. Validation by independent clinical researchers is also lacking, which may reflect concerns about their reliability or a reluctance to engage with tools that might reduce interaction with patients.

## WHAT MAKES CHATGPT AND GPT-4 UNIQUE?

The current version of ChatGPT is based on generative pretrained transformer 3 (GPT-3). OpenAI trained the model using reinforcement learning from human feedback, adapting a similar but smaller language model called InstructGPT [32]. The process by which the model was trained and fine-tuned was recently outlined [33]. Details for GPT-4 are lacking.

**Figure 1.**
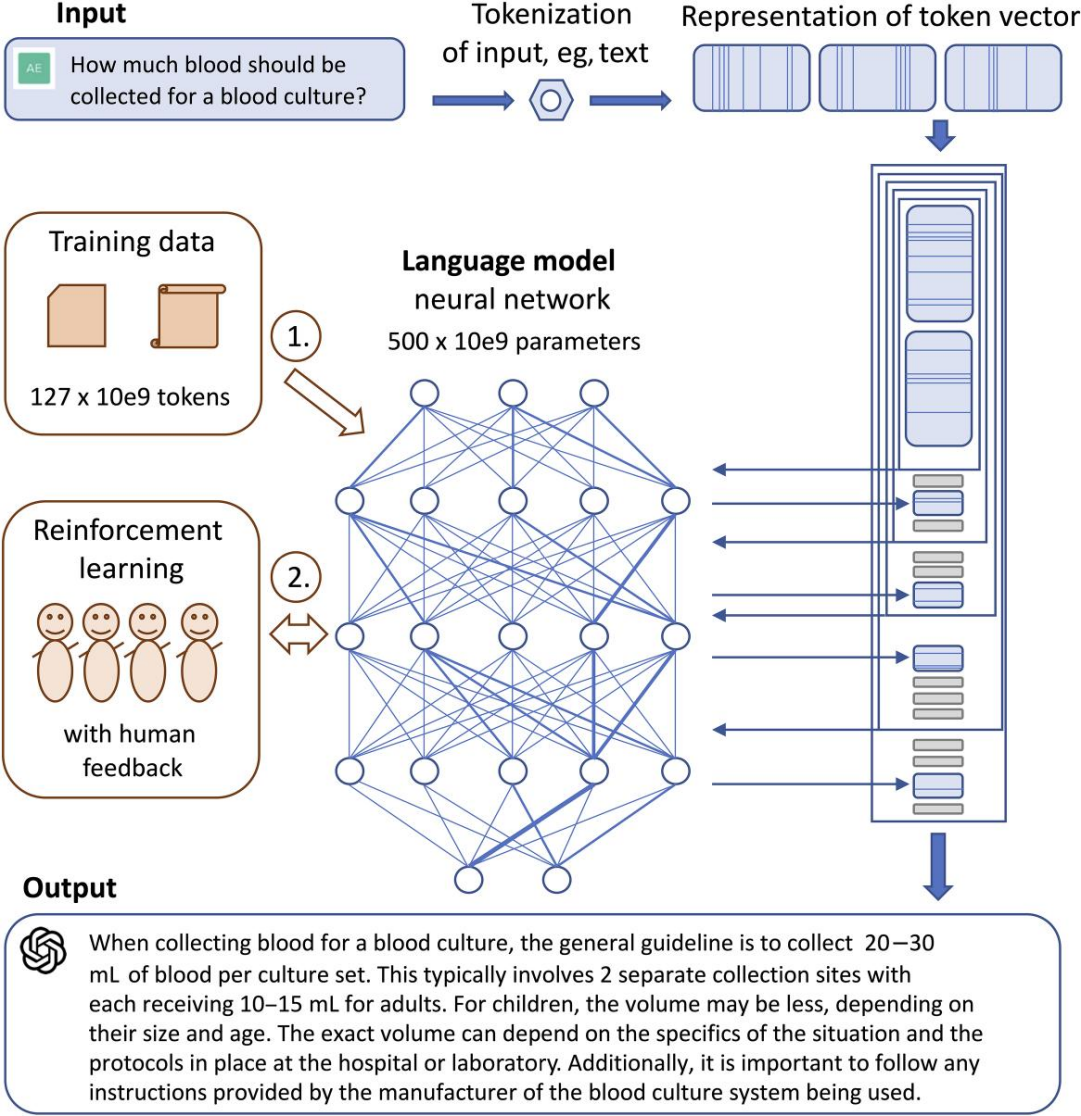
Learning and optimization steps of ChatGPT.

More than 175 billion text fragments (tokens) were integrated into the initial version of ChatGPT, although details of which sources were used for training are not publicly available (see Figure 1). ChatGPT has not been trained specifically as a medical chatbot, and it is unclear what scientific literature was accessed. That said, LLMs can cover a wide range of infectious disease and medical microbiology topics. Supplementary Table 4 provides examples of medical microbiology output and shows how answer and reference functions have evolved over the past 5 months (Supplementary Table 4).

Detailed assessment of the impact of different versions is lacking. For diagnostics, digital control questions (ie, internal controls) may help to benchmark and quality control outputs over time. Overall, the content of replies has changed in quality, and the output can be rapidly reformulated, expanded, and summarized with specific instructions. The chatbot also performs well in simplifying complex topics for lay audiences. Although reference functions have improved since launch, further work is required; ChatGPT provides entirely fabricated references and, even when asked for specific PubMed links, references completely different topics.

When using search engines for health-related questions, people are served information as a ranked index of websites rather than as processed knowledge that provides a clear, comprehensive answer to a question. Interactive NLP-based models may thus rapidly replace much of the work we rely on search engines to perform. Thanks to its application programming interface (API) and plugin function, which supports easy integration into software, we can expect LLMs to become widely adopted and integrated into a range of healthcare devices.

## REQUIREMENTS FOR INTEGRATING CHATBOTS INTO THE LABORATORY

Digital technologies are applied to a range of data types to improve the automation, efficiency, accuracy, and effectiveness of laboratory processes [1]. In a clinical microbiology laboratory, the primary source of information comes from diagnostic devices (eg, matrix-assisted laser desorption/ionization time-of-flight mass spectrometry, plate readers, sequencers), laboratory information management systems, and electronic medical records systems. Laboratory datasets and workflows can be combined into a laboratory data warehouse (LDWH) that streamlines sample collection, testing, data structure, and result reporting, covering all aspects of the pre- to post-analytical workflow [34].

Digitalization can support automation; improve the accuracy, reliability, and sensitivity of diagnostic tests; and reduce the risk of errors, thereby improving diagnostic quality. This relies on well-curated, structured, interoperable, standardized data formats that can be achieved using a laboratory ontology such as LOINC or SNOMED CT [35]. Version tracking of data and associated tools and the adoption of data interface standards that enable transfer of structured data between platforms are vital [36]. Unstructured data (eg, free-form text, images, audio, and video) that are not organized into standardized formats and may be difficult to interpret can also be of value for diagnostics but often require specialized software and machine learning techniques.

The integration of standard operating procedures, analytic tools, data types, and quality controls into an LDWH accessed by any LLM will create exciting new possibilities for improving clinical microbiology laboratory practices.

## WHERE TO USE CHATBOTS?

Multiple opportunities to integrate LLMs into the pre- to post-analytical process can be envisaged, and many more will become clear as the technology develops further. Chatbots used in healthcare must follow a tight regulatory framework that will need to be regularly adapted to recent advances in the field (see Supplementary Text 1).

### Pre-analytics

Basic pre-analytic information can be summarized in a registry of frequently asked questions. Typically, interactions between clinicians and laboratory personnel provide guidance for available diagnostic tests. A series of pre-analytical use cases for a chatbot to improve these interactions can be envisaged (Table 1). Of note, this reasonably comprehensive list was generated by ChatGPT. Applications that optimize the selection, timing, and interpretation of diagnostics to ensure that they are used appropriately are clearly attractive and could help to improve patient care.

**Table 1.** List of Pre-Analytical Applications for a Chatbot

Question	A Chatbot Application Could…
Are the patient's clinical history and current symptoms relevant to the suspected infection?	… improve the current workflow by allowing clinicians to input relevant patient information and symptoms easily and quickly, which could help guide the selection of the appropriate specimen and testing method.
Is the specimen of sufficient volume and quality for the desired tests?	… provide guidance on the appropriate volume and quality of the specimen needed for the desired tests and alert laboratory staff if the specimen does not meet these criteria.
Is the appropriate specimen being collected for the suspected infection?	… ensure that the appropriate specimen is being collected by providing guidance on the specific type of specimen that is needed for the suspected infection and the proper collection method.
Is the specimen being collected and transported in the proper manner to maintain specific integrity?	… ensure that the specimen is being collected and transported in the proper manner by providing guidance on how to handle and transport the specimen to maintain its integrity.
Is the specimen being transported to the laboratory in a timely manner?	… improve the timeliness of specimen transport to the laboratory by providing reminders and alerts to clinicians to ensure that specimens are delivered in a timely manner.
Is the correct microbiological testing method being used for the suspected infection?	… assist in selecting the appropriate microbiological testing method by providing guidance based on the patient's clinical history and symptoms, as well as the suspected infections.
Are all necessary quality-control measures being taken to ensure the accuracy of the testing?	… improve the accuracy of testing by providing reminders to laboratory staff to perform necessary quality-control measures.
Is the laboratory properly equipped and staffed to perform the testing?	… assist in ensuring that the laboratory is properly equipped and staffed by providing reminders and alerts for equipment maintenance and staff training.
Is the laboratory following the proper procedures for handling and processing the specimen?	… improve the handling and processing of the specimen in the laboratory by providing guidance on the proper procedures to follow.
Is the laboratory using the most up-to-date methods and technology for microbiological testing?	… assist in keeping the laboratory up to date with the latest methods and technology by providing alerts for updates and new technology.
Are there any potential interferences or contaminants that could affect the accuracy of the test results?	… identify potential interference or contaminants that could affect test results by providing alerts and reminders to laboratory staff to consider these factors during testing.

This list was generated by ChatGPT (GPT-3.5) by telling it to “provide a list of the most important pre-analytical questions and the application of how chatbots could help to solve them.” The output was only slightly adapted with human inputs.

### Analytics

LLMs can be used in various ways during analytical steps in developing new diagnostic assays, providing growth media recipes for rare pathogens, troubleshooting failed polymerase chain reaction (PCR) assays, designing PCR primers, and developing programming code. They can also support the interpretation of data, especially for rarely encountered pathogens. Furthermore, LLMs can report on quality control and benchmarking by analyzing laboratory performance data. Outputs of OpenAI tools have improved since launch (Supplementary Table 4), and concrete use cases can now be envisaged (Supplementary Text 3). GPT-4 is used to analyze data by providing SQL and R scripts (Supplementary Figure 1), allows access to statistical software tools (eg, Wolfram alpha), and can execute calculations and integrate results into its responses. Clearly, at this stage, no commercial LLM should be used to analyze patient histories owing to data privacy issues and lack of regulatory approval.

### Post-analytics

Communicating results to physicians and healthcare workers is an important part of the laboratory workflow. LLMs could support this process by summarizing laboratory reports (eg, key findings, abnormal findings, notes on uncommon pathogens, advice on antibiotic selection, and next steps in testing), providing an interactive user experience through real-time questions about laboratory results, sharing information on assay performance, supporting communication with patients and families and empowering patient-led decisions through a better understanding of laboratory findings, and helping the administrative workload by communicating with customers and providing evidence during price negotiations.

### Beyond Routine Diagnostics

As shown with Alpha-fold, LLMs can predict protein structures [37]. More recently, an LLM that could be scaled to 15 billion parameters was used to directly infer the full atomic-level protein structure from the primary sequence, representing an order-of-magnitude improvement in high-resolution structure prediction that should enable high-throughput structural characterization of proteins predicted from metagenomic data [38]. Another study described a language model (ProGen) that can generate protein sequences with predictable functions across large protein families. This model was trained on 280 million protein sequences from more than 19 000 families and is augmented with tags that specify protein properties [39].

LLMs can potentially be used to analyze genomic data. One approach used deep-learning methodologies adopted from NLP algorithms to model “gene semantics” based on more than 360 million microbial genes within their genomic context. The language models were used to predict functional categories for 56 617 genes and show approximately 1300 genes putatively associated with defense systems; 98% were inferred correctly [40]. In another study, genome-scale language models were used to learn the evolutionary landscape of 1.5 million severe acute respiratory syndrome coronavirus 2 genomes and accurately and rapidly identify variants of concern [41].

## CHALLENGES FOR ADOPTION OF CHATBOTS

While ChatGPT, GPT-4, and Bard are capable of providing value across a range of use cases, important challenges and limitations remain to be addressed to improve their usability and uptake in clinical microbiology laboratories.

### Nontrusted Output

Chatbots are not sufficiently mature to formally diagnose patient conditions or replace health professionals. LLMs occasionally produce outputs that are coherent but incorrect or nonsensical. For clinical microbiology and infectious diseases, LLM outputs are of good quality but without identifiable sources and references, they cannot be trusted. No sole reference is used during reinforcement learning training; rather, a multitude of different texts are used within a response. A potential solution may be a similarity analysis that provides a quality score of “matching” toward selected references specific for a given field of specialization. Although difficult to design, the introduction of automated internal and external quality-control standards will also be important to benchmark outputs across versions.

### Data Bias

LLMs require large amounts of training data to handle the complexity of natural language and need to be continuously updated and monitored for biases. It is unclear how many clinical microbiology articles were used in training ChatGPT or how up-to-date the literature was. Another potential bias might stem from institutions in high-income countries that tend to publish greater quantities and in more open formats, which may influence model training toward certain types of research and policies. Identification of training and validation data sources is important to build trust in a tool, making the black-box nature of LLMs worrisome. Although GPT-4 can access the Internet and recent medical literature (via plugins such as ScholarAI), its biases remain unclear. In addition, for some publishers, the ScholarAI plugin can only reach content from open-access journals.

### Weak Input

Most LLMs are responsive to changes in the phrasing of input, and the accuracy or comprehensiveness of an answer can be improved by rephrasing a question. For ChatGPT, the model is also prone to redundancy, excessively using certain words and phrases (ie, repeating that it is a language model developed by OpenAI). These problems stem from biases in training, where trainers favor longer, more complete answers, and result from overoptimization issues [42, 43].

### Restricted Output

ChatGPT is currently limited to providing answers that are about a quarter page long. Generation of longer coherent answers will likely require more computation capacity, as indicated by GPT-4. OpenAI also prevents their tools from responding to inappropriate requests that may not always be ideal given that it relies on subjective opinions of OpenAI and its human trainers. Tolerating a spectrum of cultural and viewpoint diversity may be critical in training LLMs. OpenAI uses a Moderation API to flag potentially dangerous content (eg, violence, self-harm, hate, and sexual content). However, this overly restricts the outputs, and a valid question might violate the chatbot's restrictions if the context and intention of the user are unclear. For example, information being sought about sexually transmitted diseases or the side effects of drugs might be wrongly flagged.

### Misuse of ChatGPT

Unfortunately, every powerful technology can be misused to harm others. It will become increasingly important to provide training in the responsible use of AI technologies at all levels of education and society. For example, ChatGPT can write convincing scientific abstracts based on completely imaginary data that may pose challenges to detection [44]. Distinguishing AI- and human-generated content is not easy but will be important when it comes to copyright infringement and plagiarism. Furthermore, whether the individual using the tool, the tool’s owner, or the individuals whose content was used to create the output own the copyright is a thorny problem. Some tools identify AI-generated content such as GPTzero [45], GPT-2 Output Detector [46], and Writer [47]. These concerns may eventually seem relatively minor compared with other envisaged misuses. LLMs have the potential to provide instruction and code to hack computer systems with sensitive healthcare information or to generate disinformation.

## WHAT ARE THE NEXT STEPS?

Use of chatbots in microbiological diagnostics remains in its infancy. However, the launch of ChatGPT likely represents a defining moment in the appreciation and adoption of AI-based technologies across a range of sectors. Its language model opens new capabilities for various fields and will likely have a substantial impact on medicine.

Several steps will be critical for this technology to become routine in microbiology diagnostics. Guidelines for chatbot use are needed from professional societies, and the applications that are developed should integrate the pre- to-post analytical understanding of diagnostic workflows. In a recent opinion paper on the requirements for implementation of GPT-3 chatbots in healthcare, the authors included processing needs and information systems infrastructure, operating costs, model biases, and evaluation metrics [48]. They identified 3 major operational factors that may drive the adoption: ensuring Health Insurance Portability and Accountability Act compliance, building trust with healthcare providers, and establishing broader access to the GPT-3 tools.

AI-generated outputs need to be monitored and validated by independent researchers and medical experts. Evaluation using RCTs and other types of trials is needed to demonstrate reliability and build trust. Furthermore, AI outputs should be clearly labeled, and a standard for citation of AI-generated content should be developed (eg, a digital watermark). Safety practices for AI-based content are needed to help identify fabricated scientific content. Ultimately, the technology may follow the trajectory of the smartphone, reaching a level where every individual carries with them a personal chatbot that is used in all aspects of daily life. Such “digital avatars” may be trained by any content a person produces (eg, email, messages, text, and audio output) and may even process information and communicate with doctors regarding medical issues or behavioral changes, bringing challenges with data security and privacy that will need to be considered.

The possibilities for LLMs and subsequent AI-based technologies are tremendous and are being realized very fast. Their adoption should be encouraged. However, we should be aware of the challenges and threats, addressing them with transparency and through education on the appropriate and inappropriate use of such tools. During the next 5–10 years, we will encounter profound technological developments in the fields of machine learning and AI that will impact our daily work at the routine laboratory in yet unimaginable ways. Make educating yourself about AI a priority.

## Supplementary Data

Supplementary materials are available at *Clinical Infectious Diseases* online. Consisting of data provided by the authors to benefit the reader, the posted materials are not copyedited and are the sole responsibility of the authors, so questions or comments should be addressed to the corresponding author.

## Supplementary Material

ciad407_Supplementary_Data

## Notes

***Acknowledgments.*** The author thanks ChatGPT and GPT-4 (OpenAI LLC, 3180 18th Street, San Francisco, California) for their valuable input in writing this article. There is a debate on whether chatbots should be included as a coauthor. According to International Committee of Medical Journal Editors guidelines, a coauthorship was not justified, and ChatGPT and GPT-4 (as any current LLM) cannot consent and take responsibility for the text. Citations generated by ChatGPT or GPT-4 are clearly marked in the text. Special thanks to my human colleagues, especially Dr Frank Imkamp, Dr Aline Cuénod, Dr Fanny Wegner, and Zoja Germuskova (Institute of Medical Microbiology, University of Zurich), for critical reading and valuable discussions during the writing and editing process. Special thanks to Dr Andy Jermy from Germinate Science Consulting for editing the manuscript.
