# Supplementary material for: ChatGPT, GPT-4, and Other Large Language Models: The Next Revolution for Clinical Microbiology?
Source: Clin Infect Dis. 2023 Jul 3;77(9):1322–8. doi: 10.1093/cid/ciad407 (PMC10640689; doi:10.1093/cid/ciad407)
Supplement: ciad407_Supplementary_Data [file ciad407_supplementary_data.zip › Supplementary Table 1.docx]

**Supplementary Table 1.** **Selected LLMs launched since 2022.**

| **LLM** | **Company** | **Application** | **Reference** |
| --- | --- | --- | --- |
| ChatGPT | OpenAI | AI chatbot | <https://openai.com/chatgpt> |
| GPT-4 | OpenAI | AI chatbot | <https://openai.com/research/gpt-4> |
| Bard | Google | AI chatbot | <https://bard.google.com/> |
| Claude | Anthropic | AI chatbot | [www.anthropic.com](http://www.anthropic.com) |
| Jasper | Jasper | AI writing tool designed to generate marketing copy, such as blog posts, product descriptions, company bios, ad copy | <https://www.jasper.ai/> |
| Writesonic | Writesonic | AI writer that creates SEO-friendly content for blogs and ad copy | <https://writesonic.com> |
| You | You.com | AI-powered search engine | htttps://you.com |
