# Supplementary material for: ChatGPT, GPT-4, and Other Large Language Models: The Next Revolution for Clinical Microbiology?
Source: Clin Infect Dis. 2023 Jul 3;77(9):1322–8. doi: 10.1093/cid/ciad407 (PMC10640689; doi:10.1093/cid/ciad407)
Supplement: ciad407_Supplementary_Data [file ciad407_supplementary_data.zip › Supplementary Table 2.docx]

**Supplementary Table 2.** **Algorithm elements of a natural language processing (NLP)-based model.** ChatGPT (GPT-3.5) was used to provide these definitions and verified with the literature.

| **Task** | **Definition** |
| --- | --- |
| Language recognition | The ability to identify and classify the language being used by a speaker or writer. The language of the input must be correctly identified to apply the appropriate language-specific processing. Language recognition models typically use a combination of techniques to identify the language of a given input. These include:   - Character n-grams: This method involves analyzing the frequency of combinations of characters (n-grams) in the input. Different languages have distinct character n-gram distributions, which can be used to identify the language. - Dictionary-based: This method involves comparing the words in the input to a dictionary of words from different languages. The language with the highest number of matching words is considered the most likely language of the input. - Machine learning: This method involves training a machine learning model on a large dataset of labeled text samples in different languages. The model can then be used to classify new text samples based on their language. - Neural Networks: This method involves using neural networks like Long Short-Term Memory (LSTM), Gated Recurrent Unit (GRU) and transformer to identify the language of the input. |
| Tokenization | The process of breaking down a piece of text into smaller units (token), such as words, subwords, or phrases. There are several different tokenization methods that can be used in NLP, including:   - Word tokenization: This method breaks down the text into individual words. This is the most common form of tokenization and is used in many NLP tasks, such as language modeling and text classification. - Subword tokenization: This method breaks down words into smaller units called subwords. The main reason to use subword tokenization is that it can handle out-of-vocabulary words, which are the words that the model has not seen in the training data. This is achieved by breaking down the words into smaller units, and then the model can learn to compose the subwords to generate a new word. - Sentence tokenization: This method breaks down the text into individual sentences. This is useful for tasks such as text summarization and machine translation, where the model needs to work with individual sentences. - Character tokenization: This method breaks down the text into individual characters. This is useful for tasks such as handwriting recognition and text generation. |
| Part of speech (POS) tagging | The process of identifying the POS of each word in a piece of text (e.g., noun, verb, or adjective). POS provides more structural and grammatical information about the text. This information can be used to improve the performance of other NLP tasks, such as syntactic parsing, semantic role labeling, and named entity recognition. There are different POS tagging techniques that can be used in NLP, including:   - Rule-based tagging: This method uses a set of predefined rules and patterns to assign POS tags to words. The rule-based approach is generally considered to be less accurate than the other techniques. - Statistical tagging: This method involves training a machine learning model on a large corpus of text that has been pre-annotated with POS tags. The model is then used to predict the POS tags for new text. This approach is generally considered to be more accurate than the rule-based approach. - Hybrid tagging: This method combines both rule-based and statistical approaches, by using a set of predefined rules to assign POS tags, and then using a machine learning model to correct any errors made by the rules. |
| Name entity recognition (NER) | The process of identifying and classifying named entities in a piece of text e.g., people, organizations, locations, or diseases. It is a subtask of information extraction that seeks to locate and classify named entities in text into predefined categories such as person names, organizations, locations, medical codes, time expressions, quantities, monetary values, percentages, etc. NER systems can be based on rule-based, statistical or a combination of both methods.   - Rule-based NER systems rely on predefined rules and patterns to identify named entities. These systems are typically less accurate than statistical systems and require a significant amount of human effort to develop and maintain. - Statistical NER systems, on the other hand, use machine learning algorithms to learn to recognize named entities from a large, annotated corpus of text. These systems are generally more accurate than rule-based systems and can be easily adapted to new domains and languages. - Hybrid systems combine the strengths of both rule-based and statistical systems, by using a set of predefined rules to identify named entities, and then using a machine learning model to correct any errors made by the rules. |
| Sentiment analysis | The process of determining the emotional tone or sentiment of a piece of text. It is used to determine the attitudes, opinions, and emotions of a speaker or writer with respect to some topic or the overall contextual polarity of a document. Sentiment analysis can be applied to a variety of sources. There are several different techniques that can be used for sentiment analysis, including:   - Dictionary-based: This method involves comparing words in the text to a pre-defined dictionary of words that are associated with positive or negative sentiment. - Machine learning: This method involves training a machine learning model on a large dataset of labeled text samples, where the sentiment of the text has been pre-determined. The model can then be used to classify new text samples based on their sentiment. - Neural networks: This method involves using neural networks to analyze the text and identify patterns that indicate sentiment.   Sentiment analysis is a challenging task, as human language is often ambiguous and context-dependent, and the same word can have different sentiment depending on the context. Additionally, it is important to consider the cultural and historical context of the text, as the same words or expressions can have different meanings depending on the culture. |
| Topic modelling | An unsupervised NLP technique to tag and group text clusters that share common topics e.g., diagnostics, therapy, and prevention. The main idea behind topic modeling is to identify patterns in the occurrence of words within a corpus of text and group them into topics. These topics can then be used to summarize the content of the text, classify it into different categories, or even generate new text. There are several popular topic modeling techniques, including:   - Latent Dirichlet Allocation (LDA): This is one of the most widely used topic modeling techniques. It is a generative probabilistic model that assumes that each document is a mixture of a small number of latent topics. - Latent Semantic Analysis (LSA) : This technique is based on the idea that words that are close in meaning will occur in similar contexts. It uses linear algebra to identify patterns in the co-occurrence of words in a corpus of text. - Non-negative Matrix Factorization (NMF): This is a linear algebraic technique that is similar to LSA and is used to find the underlying topics in a corpus of text. |
| Machine translation | The process of translating text from one language to another e.g. DeepL ([www.deepl.com](http://www.deepl.com)). There are two main approaches to machine translation: rule-based and statistical.   - Rule-based machine translation (RBMT) relies on a set of predefined rules and linguistic knowledge to translate text. The main advantage of RBMT is that it can produce grammatically correct translations, but its main disadvantage is that it is limited by the quality and coverage of the linguistic knowledge it relies on. - Statistical machine translation (SMT) uses statistical models to translate text. These models are trained on large parallel corpora of text, which consist of sentences or paragraphs in different languages that have been translated by humans. The models learn patterns in the data and use them to generate translations. The main advantage of SMT is that it can handle large amounts of data and can adapt to different domains and languages. |
| Text summary | Breakdown of text or jargon into its most basic terms using NL processing to make it more understandable. There are two main approaches to text summarization: extractive and abstractive.   - Extractive summarization: This method involves selecting the most important sentences or phrases from the original text and concatenating them to form a summary. This approach is based on the idea that the most important information in a text is contained in individual sentences or phrases. Extractive summarization is more accurate than abstractive summarization but can sometimes produce summaries that are not fluent or natural-sounding. - Abstractive summarization: This method involves generating a new summary that is not directly based on the original text. This approach is based on the idea that the most important information in a text can be inferred by understanding the meaning of the text. Abstractive summarization is less accurate than extractive summarization but can produce summaries that are more fluent and natural-sounding.   Recently, neural network-based models such as transformer-based architectures have been proposed for text summarization, which have been found to produce more coherent and natural summaries. |
