# Supplementary material for: ChatGPT, GPT-4, and Other Large Language Models: The Next Revolution for Clinical Microbiology?
Source: Clin Infect Dis. 2023 Jul 3;77(9):1322–8. doi: 10.1093/cid/ciad407 (PMC10640689; doi:10.1093/cid/ciad407)
Supplement: ciad407_Supplementary_Data [file ciad407_supplementary_data.zip › Supplementary Table 3.docx]

**Supplementary Table 3.** **Selection of medical chatbots and symptom checkers** (accessed via PubMed and Google search). *, ChatGPT and GPT-4 is not considered a medical chatbot also not FDA-approved at this stage. However, this type of LLM allows to answer a broad range of general medical questions and it has also passed board exams in medicine (**reference**). Often no published technical information exists and the content on the website of the company was cited.

| **Name** | **Company** | **Release** | **Application** | **Reference** |
| --- | --- | --- | --- | --- |
| Ada | Ada Health, Berlin, Germany | 2017 | Health chatbot that uses “AI” to provide personalized health information and recommendation to users. Users can input their symptoms and medical history. Ada will provide a list of possible conditions and suggests next steps for seeking medical attention. | https://ada.com/de/ |
| HealthTap | HalthTap, Sunnydale, USA | unknown | A chatbot that allows users to ask health-related questions and receive answers from a network of licensed doctors.  “HealthTap has the country's largest network of volunteer doctors across 140+ specialties to create a database of millions of trustworthy doctor answers. ” | https://www.healthtap.com/ |
| Woebot | Woebot Health | 2017 | «Personal mental health ally that helps you get back to feeling like yourself. Grounded in science and powered by AI, Woebot is easy to talk to and fits right into your life, whenever you want to chat.» | https://woebothealth.com/ |
| Healthily | Your.MD | 2015 | Chatbot that provides information on health conditions, medications, and treatments, as well as support for managing chronic conditions such as diabetes and hypertension. | https://www.livehealthily.com/ |
| ChatGPT/GPT-4* | OpenAI | 2022 | A LLM and reinforcement learning boosted model. | https://openai.com/ |
