# Supplementary material for: ChatGPT, GPT-4, and Other Large Language Models: The Next Revolution for Clinical Microbiology?
Source: Clin Infect Dis. 2023 Jul 3;77(9):1322–8. doi: 10.1093/cid/ciad407 (PMC10640689; doi:10.1093/cid/ciad407)
Supplement: ciad407_Supplementary_Data [file ciad407_supplementary_data.zip › Supplementary Table 4.docx]

**Supplementary Table 4. Evolution of ChatGPT and GPT-4 outputs on the same question over five months.** One examples of a question asked to GPT-3.5 (Dec 15th, 2022), GPT-3.5 (May 12th, 2023), and GPT-4 (May 12th, 2023) on a topic related to medical microbiology. The table shows the prompt, the response, and an expert opinion.

| **Prompt** | **Answer from ChatGPT** | **Reference** | **Expert evaluation of response** |
| --- | --- | --- | --- |
| **GPT-3.5 (Dec 15th 2022)**  “Write a short summary about the differences between S. aureus and S. epidermidis.”  “Please provide the proper Pubmed reference.” | “Staphylococcus aureus and Staphylococcus epidermidis are two species of gram-positive bacteria that can cause a range of infections in humans. While both species are commonly found on the skin and mucous membranes, S. aureus is more likely to cause infections and is more resistant to antibiotics compared to S. epidermidis. S. aureus is a more virulent pathogen and can cause a wide range of infections, including skin infections, respiratory infections, and food poisoning. In contrast, S. epidermidis is typically associated with less severe infections such as skin infections and infections related to indwelling medical devices, and is generally more sensitive to antibiotics.” | “Wang, Y., & Lu, Y. (2014). Staphylococcus aureus and Staphylococcus epidermidis: differences and similarities. Frontiers in Microbiology, 5, 677.” | The content of the short reply is proper, but the reference does not exist. |
| **GPT-3.5 (May 12^th^ 2023)**  “Write a short summary about the differences between S. aureus and S. epidermidis.”  “Please provide the proper Pubmed reference.” | “Staphylococcus aureus and Staphylococcus epidermidis are two commonly encountered bacteria in human microbiota, but they differ in their characteristics and clinical significance. S. aureus is a pathogenic bacterium that can cause a range of infections, including skin and soft tissue infections, pneumonia, endocarditis, and sepsis. It produces numerous virulence factors, such as toxins and enzymes, which contribute to its pathogenicity. S. aureus is often resistant to antibiotics, including methicillin-resistant Staphylococcus aureus (MRSA), which poses a significant healthcare challenge.  On the other hand, S. epidermidis is typically a commensal bacterium found on the skin and mucous membranes. It is considered a less virulent species compared to S. aureus. While S. epidermidis can cause infections, such as catheter-related bloodstream infections and infections in immunocompromised individuals, it is generally less aggressive. S. epidermidis is also known for its ability to form biofilms on medical devices, which can contribute to the development of device-related infections.” | “Otto M. Staphylococcus epidermidis—the 'accidental' pathogen. Nat Rev Microbiol. 2009 Oct;7(8):555-67. doi: 10.1038/nrmicro2182. PMID: 19561604.” | The content of the short reply is corret, the reference as well, but the provided PMID number is wrong. |
| **GPT-4 (May 12th 2023)**  “Write a short summary about the differences between S. aureus and S. epidermidis.”  “Please provide the proper Pubmed reference.” | “Staphylococcus aureus and Staphylococcus epidermidis are both Gram-positive bacteria.  S. aureus is often pathogenic and can cause a variety of infections, including skin infections, pneumonia, food poisoning, and severe systemic infections. It is coagulase-positive, meaning it produces an enzyme, coagulase, that clots blood plasma. S. aureus is also known to produce a variety of toxins.  On the other hand, S. epidermidis is coagulase-negative and is generally less virulent than S. aureus. It is a significant component of the skin microbiota and usually only causes infections in compromised hosts or when it has access to the normally sterile parts of the body through medical devices. Its primary pathogenic trait is its ability to form biofilms.  For more detailed and updated information, I recommend directly checking peer-reviewed articles or reviews from PubMed or similar databases.” | “As an AI, I'm unable to directly fetch or generate specific references. However, the information provided is standard microbiology and can be found in many textbooks and review articles. One such source is the following:  Title: "Medical Microbiology. 4th edition." Authors: Baron S, editor. Publisher: Galveston (TX): University of Texas Medical Branch at Galveston; 1996. Chapter 12: "Staphylococcus" Available from: <https://www.ncbi.nlm.nih.gov/books/NBK8448/>  Please consult this and other similar resources for more detailed information and context. For the most recent data and research, you would need to check the latest articles on PubMed or another scientific database.” | “The content is correct and also the reference is correct.” |
