# Supplementary material for: ChatGPT, GPT-4, and Other Large Language Models: The Next Revolution for Clinical Microbiology?
Source: Clin Infect Dis. 2023 Jul 3;77(9):1322–8. doi: 10.1093/cid/ciad407 (PMC10640689; doi:10.1093/cid/ciad407)
Supplement: ciad407_Supplementary_Data [file ciad407_supplementary_data.zip › Supplementary Text 1.docx]

**Supplementary Text 1.** How are Large Language Models trained and what are the technical requirements?

ChatGPT and GPT-4 are examples of LLMs using a transformer, which has been previously trained on a large dataset, hence the name pre-trained transformer model (1). Such models work based on the mechanism of attention, identifying interactions between words (tokens) in sentences. It can process the entire sentence simultaneously, making it highly efficient for parallel computation. Usually, these models are trained unsupervised on a large corpus of text data (2), but this technique can also be used to explore e.g. transcriptomics data (3). The pre-training process involves learning to predict a word in a sentence given the other words (a task called “masked language modeling”), or learning to predict the next sentence (a task called “next sentence prediction”) (4). After the pre-training phase, these models can be fine-tuned on a specific task such as text classification, named entity recognition, sentiment analysis, etc., with less training data. The fine-tuning process leverages the model’s knowledge during pre-training, leading to good performance even with smaller task-specific datasets. Examples of pre-trained transformer models include BERT (Bidirectional Encoder Representations from Transformers, (4)), GPT (Generative Pretrained Transformer; (5)), and others.

To generate such powerful LLMs three important requirements must be fulfilled: (i) Neural networks, (ii) computational power, and (iii) a large amount of data.

- *Neural network.* Today’s neural networks used in LLMs encompass up to 1 trillion parameters, which is a surrogate of the numbers of connections between neurons. This provides the ability to calculate highly complex tasks.
- *Computational power.* The required computational power can be expressed as floating-point operations (flop), which corresponds to the number of arithmetic calculations a system needs to perform. For this usually graphic processing units (GPUs) are used which provide today on average around 15 terra flops per second (10e12/sec). LLMs such as GPT-4 however required around 1000 zeta flops of calculations (10e24 flops) (6, 7). This also explains the company dependence such LLMs generate, as several 1000 GPUs are required to keep the training period below one year
- *Large amount of data.* As discussed in the main part of the article ChatGPT and GPT-4 used large amounts of text. As explained in **Supplementary Table 2**, the text is usually fragmentized into individual tokens. A token is a piece of information e.g., a word in a sentence or a pixel in an image. ChatGPT supposedly used 175 billion tokens for training. However, the exact text files which were used for training are unknown and therefore also the bias cannot be estimated.

Interestingly, newer computation cheaper models were released such as Standford’s Alpaca. This model is based on Meta’s Llama model – a 8 billion parameter model. An API to ChatGPT has been used to train Standford’s Alpaca and it reached very good overall performance (8). In the future, likely we will see more examples where AI will train AI. This poses also very important regulatory questions on how AI should be allowed to interact with other AI.

*References*

1. Vaswani A, Shazeer N, Parmar N, Uszkoreit J, L. J, Gomez AN, et al. Attention Is All You Need. arXiv. 2017:15.

2. Doersch C, Zisserman A. Multi-task self-supervised visual learning. Arxiv. 2017;arXiv:1708.07860.

3. Long Y, Ang KS, Li M, Chong KLK, Sethi R, Zhong C, et al. Spatially informed clustering, integration, and deconvolution of spatial transcriptomics with GraphST. Nat Commun. 2023;14(1):1155.

4. Devlin J, Chang M-W, Lee K, Toutanova K. BERT: Pre-training of Deep Bidirectional Transformers for Language Understanding. Arxiv. 2018;arXiv:1810.04805

5. Brown TB, Mann B, Ryder N, Subbiah M, Kaplan J, Dhariwal P, et al. Language Models are Few-Shot Learners. Arxiv. 2020.

6. Sevilla J, Heim L, Ho A, Besiroglu T, Hobbhan M, Villalobos P. Compute Trends Across Three Eras of Machine Learning. Arxiv. 2022.

7. Ananthaswamy A. In AI, is bigger always better? Nature. 2023;615(7951):202-5.

8. Wijeratne Y, Marikar I. Better Question - Answering Models on a Budget. arxiv. 2023.
