# Supplementary material for: ChatGPT, GPT-4, and Other Large Language Models: The Next Revolution for Clinical Microbiology?
Source: Clin Infect Dis. 2023 Jul 3;77(9):1322–8. doi: 10.1093/cid/ciad407 (PMC10640689; doi:10.1093/cid/ciad407)
Supplement: ciad407_Supplementary_Data [file ciad407_supplementary_data.zip › Supplementary Text 2.docx]

**Supplementary Text 2. The regulatory framework.**

Although this opinion paper is not focusing on the regulatory aspects of AI in medicine, the most important aspects are summarized below.

Regulation of chatbots and symptom checkers as medical devices varies significantly between jurisdictions, with different approaches in the United States and the European Union (1). In the US, the FDA classifies these tools under the Medical Device Amendment of 1976 (2, 3). Once they receive clearance as in vitro diagnostic (IVD) devices, they are 'locked' from further algorithmic learning to maintain the state for which they were approved (107). This poses a significant challenge, as one of the core advantages of AI systems is their ability to continually learn and adapt from new data.

Contrastingly, the EU follows the General Data Protection Regulation (GDPR) and Medical Devices Regulation (MDR), which mandate transparency and consent, and a 'right to explanation' for algorithmic decisions affecting users (4, 5, 6). Under this framework, AI devices must provide understandable information about their logic, significance, and consequences. These requirements, while fostering transparency, can be at odds with the inherently opaque nature of complex AI systems.

Three key regulatory issues arise from this comparison:

1. How can regulators permit continuous learning in AI while ensuring the consistency and safety of its applications in healthcare?
2. How to balance the need for transparency and the 'right to explanation' against the complex and often 'black box' nature of AI?
3. How can regulators ensure rigorous, independent validation and benchmarking of AI tools while encouraging innovation and progress in this field?

Despite these challenges, a variety of chatbots and symptom checkers are available and are being used in medical applications. However, as this field grows, a more flexible and AI-tailored regulatory framework will be required, one that balances patient safety, privacy, and the potential of AI. In general, some of the regulatory documents and law lag the fast technological evolution.

Another interesting regulatory aspect is what technology user accept in terms of risk taking e.g., in delegation of decisions (7).

*References*

1. Vokinger KN, Hwang TJ, Kesselheim AS. Lifecycle Regulation and Evaluation of Artificial Intelligence and Machine Learning-Based Medical Devices. In: Shachar C, Robertson C, Cohen IG, Minssen T, Price Ii WN, editors. The Future of Medical Device Regulation: Innovation and Protection. Cambridge: Cambridge University Press; 2022. p. 13-21.

2. Administration USFaD. Artificial Intelligence and Machine Learning (AI/ML)-Enabled Medical Devices 2022 [Available from: <https://www.fda.gov/medical-devices/software-medical-device-samd/artificial-intelligence-and-machine-learning-aiml-enabled-medical-devices>.

3. Administration USFaD. Artificial Intelligence and Machine Learning (AI/ML) Sotware as a Medical Device - Action Plan 2021 [Available from: <https://www.fda.gov/medical-devices/software-medical-device-samd/artificial-intelligence-and-machine-learning-software-medical-device>.

4. Meszaros J, Compagnucci MC, Minssen T. The Interaction of the Medical Device Regulation and the GDPR: Do European Rules on Privacy and Scientific Research Impair the Safety and Performance of AI Medical Devices? In: Shachar C, Robertson C, Cohen IG, Minssen T, Price Ii WN, editors. The Future of Medical Device Regulation: Innovation and Protection. Cambridge: Cambridge University Press; 2022. p. 77-90.

5. Regulation (EU) 2017/745 on medical devices, (2021).

6. Regulation (EU) 2017/746 on in vitro diagnostic medical devices, (2021).

7. Candrian C, Scherer A. Rise of the machines: Delegating decisions to autonomous AI. Computers in Human Behavior. 2022;134:107308.
