# Supplementary material for: ChatGPT, GPT-4, and Other Large Language Models: The Next Revolution for Clinical Microbiology?
Source: Clin Infect Dis. 2023 Jul 3;77(9):1322–8. doi: 10.1093/cid/ciad407 (PMC10640689; doi:10.1093/cid/ciad407)
Supplement: ciad407_Supplementary_Data [file ciad407_supplementary_data.zip › Supplementary Text 3 - final.docx]

**Supplementary Text 3. Example analytical use cases for GPT-4.**

***Use case 1:*** *Technical trouble shooting of a failed diagnostic test.*

Prompt: “What are the potential reasons for a failed 16S PCR in a tissue sample? Provide a list of trouble shooting tips and tricks.”

GPT-4 replied as follows (prompt 18^th^ May 2023, version 12^th^ May 2023):

“Sure, PCR, including 16S rRNA gene PCR, can sometimes fail due to a variety of reasons. Here are some potential causes for a failed 16S PCR in a tissue sample:

1. **Poor quality or quantity of DNA:** If the DNA extracted from the tissue sample is degraded or insufficient, this can lead to a failed PCR. It is crucial to ensure DNA is properly extracted and stored. Also, the use of an inappropriate extraction method may lead to a loss or degradation of the DNA.
2. **Presence of PCR inhibitors:** Sometimes, tissue samples may contain substances that inhibit PCR, such as hemoglobin, heparin, polysaccharides, and certain salts. These inhibitors can prevent DNA polymerase from functioning correctly.
3. **Wrong PCR reagent concentrations:** This can include the DNA polymerase, primers, Mg2+, dNTPs, or the buffer. If the balance is off, it may not be optimal for the reaction.
4. **Inappropriate PCR cycling conditions:** If the temperature or time for the denaturation, annealing, or extension steps is incorrect, it can prevent the PCR from working.
5. **Primer issues:** Primers may not be correctly designed for the 16S rRNA gene, may not be specific enough, or may have degraded.
6. **Contamination:** Contamination with other DNA, particularly DNA that also contains the 16S rRNA gene, can cause issues with the PCR.

Here are some troubleshooting tips and tricks:

1. **Check DNA quality and quantity:** Use a spectrophotometer or fluorometer to ensure that you have enough high-quality DNA. You can also run a gel to check for DNA degradation.
2. **Check for PCR inhibitors:** Test for the presence of PCR inhibitors by adding a known amount of a different DNA to your sample and trying to amplify it. If it does not amplify, inhibitors may be present.
3. **Adjust reagent concentrations:** If you suspect the issue might be due to incorrect concentrations, you may want to perform a gradient PCR to optimize the Mg2+ concentration, or adjust the amount of enzyme, primers, or dNTPs.
4. **Optimize cycling conditions:** Consider adjusting the annealing temperature or time of the PCR cycles. Gradient thermocyclers can be useful for finding the optimal annealing temperature.
5. **Check primers:** Verify the primer sequences and redesign if necessary. Also, ensure that they are stored properly and have not degraded.
6. **Minimize contamination:** Use aseptic techniques, use negative controls to detect contamination, and consider setting up a separate area for PCR setup.

Remember, each PCR reaction can be a bit different, so it might take a few tries to find the optimal conditions.”

While the answer is rather simplistic, on each point more details can be requested, providing valuable input on where to start trouble shooting.

***Use case 2:*** *Designing primers for pathogen detection or characterization.*

When asked “I want to design some primers for a 16S sequencing project - what would you recommend - please consider primers from the V4 region of the EMP.”, GPT-4 replied (prompt 20^th^ May 2023, version 12^th^ May 2023):

“For a 16S sequencing project focusing on the V4 region, I would recommend the primers used by the Earth Microbiome Project (EMP). These primers have been extensively tested and used in a variety of projects, ensuring their robustness and efficacy.

The sequences for these primers are as follows:

- Forward primer (515F): GTGYCAGCMGCCGCGGTAA
- Reverse primer (806R): GGACTACNVGGGTWTCTAAT

In these sequences, 'Y' stands for C or T and 'N' stands for any base (A, T, C, or G). These degenerate positions help to ensure that the primers are able to bind to a wider range of 16S rRNA gene sequences, increasing the diversity of bacterial species that can be detected​^0^FWD:GTG Y CAGCMGCCGCGGTAA; REV:GGACTAC N VGGGTWTCTAAT","pub_date":null}}&#8203;&#8203;oaicite:{"index":1,"metadata":{"title":"earthmicrobiome.org","url":"[https://earthmicrobiome.org/protocols-and-standards/16s/","text":"Ordering](https://earthmicrobiome.org/protocols-and-standards/16s/%22,%22text%22:%22Ordering) primers\n\nThe primer sequences in this protocol are always listed in the 5′ -> 3′ orientation. This is the orientation that should be used for ordering. See the page Primer Ordering and Resuspension for more information. Primers and primer constructs were designed by Greg Caporaso (2011, 2012). Modifications to primer degeneracy were done by the labs of Jed Furhman (Parada et al., 2016) and Amy Apprill (Apprill et al., 2015). Forward-barcoded constructs were redesigned by Walters et al. (2016) based upon the original constructs generated by Caporaso et al. (2012). The 926R R2 sequencing primer was extended (it includes a fragment of the 3’ adapter) to increase the Tm (which the MiSeq in particular was sensitive to","pub_date":null}}`​.

The EMP also provides a detailed protocol for the PCR amplification, which I would recommend following. This includes the concentrations of primers and other components in the PCR reaction, the cycling conditions for the thermocycler, and steps for post-PCR processing like cleaning up the amplicon pool​[^3^](https://earthmicrobiome.org/protocols-and-standards/16s/)​​[^4^](https://earthmicrobiome.org/protocols-and-standards/16s/)​​[^5^](https://earthmicrobiome.org/protocols-and-standards/16s/)​.

Please note that these primers are designed to amplify the V4 region of the 16S rRNA gene, which provides good taxonomic resolution for many bacterial species. However, different regions of the 16S rRNA gene can provide different levels of taxonomic resolution, so the choice of region (and thus the choice of primers) may depend on the specific goals and constraints of your project.”

The provided primers target the conserved region of the V4 region (515F and 806R). Of note the internet access plugin was used and GPT-4 accessed the website of the Earth Microbiome Project. However, the output visualization of GPT-4 generated an odd output error.

***Use case 3:*** *Bioinformatic analysis and coding.*

ChatGPT and GPT-4 can make code suggestions in several languages e.g., R, SQL, or python. The code can be commented, expanded, shorted, improved based on the user instructions (see **Supplementary Figure 1**). This is especially helpful during learning, when not all command lines are known. ChatGPT and GPT-4 can also provide templates and helping to properly document and explain the code. Although the code suggested may not be perfect, it might save hours of work. More advanced progress in automated coding has been made with Google’s DeepMind and its AlphaCode algorithm. AlphaCode, performed competitively in simulated evaluations on recent programming competitions (1). AlphaCode solves problems by generating millions of diverse programs using specially trained transformer-based networks and then filtering and clustering those programs to a maximum of just 10 submissions.

*Reference*

1. Li Y, Choi D, Chung J, Kushman N, Schrittwieser J, Leblond R, et al. Competition-level code generation with AlphaCode. Science. 2022;378(6624):1092-7.

**Supplementary Figure 1.** The graph shows an interaction with GPT-4 to first query an SQL database with the proper code (left) and then use the queried data and visualize the output using an R graph (right).
